# A Human Body Pressure Distribution Imaging System Based on Wavelet Analysis and Resistance Tomography

**DOI:** 10.3390/s17112634

**Published:** 2017-11-15

**Authors:** Shuanfeng Zhao, Wenbo Wang, Wei Guo, Chuanwei Zhang

**Affiliations:** School of Mechanical Engineering, Xi’an University of Science and Technology, Xi’an 710054, China; 201503120@stu.xust.edu.cn (W.W.); guow@xust.edu.cn (W.G.); zhangcw@xust.edu.cn (C.Z.)

**Keywords:** pressure distribution sensor, wavelet analysis, electrical resistance tomography

## Abstract

In this paper, a pressure distribution sensing system based on wavelet analysis and resistance tomography is proposed to overcome the shortcomings of a traditional electrode type pressure distribution sensor, which needs to be arranged with many electrodes and has a high production cost. The system uses ADS1256, a constant current source module, a serial communication module, a Raspberry host, a touch screen, and other components. The wavelet transform is used to preprocess the collected signal to improve the anti-jamming performance of the system. The method of resistance tomography is used to realize the real-time imaging of pressure distribution. Finally, the reliability of the system is verified using conductive silica gel as a sensitive material. The experimental results show that wavelet analysis preprocessing can significantly improve the quality of pressure distribution imaging.

## 1. Introduction

In recent years, the measurement of human body pressure distribution has become an important problem in the research of medical, bionics, and other disciplines. Human body pressure is the pressure produced by the contact surface when the body is in contact with outside objects. The peripheral blood circulation is bad in patients with diabetes mellitus, as it is easy to form diabetic foot. The analysis of plantar dynamic pressure distribution and its morphological characteristics can accurately diagnose diabetic foot [1,2,3,4]. When a dentist diagnoses a patient’s occlusal state, it is necessary to measure the size and distribution of the occlusal force of the upper and lower teeth [5,6]. With the progress of social civilization, the proportion of mental work is increasing. The comfort of the seat is directly related to the work efficiency. Unreasonable design of the seat will make people fatigue easily, and serious cases can lead to disc herniation and other diseases. The distribution of pressure produced by the body in contact with the seat is an important means of evaluating seat comfort [7,8,9]. For the paraplegic, the long-term compression of the body will cause pressure sores if effective measures to reduce the pressure are not taken. This disease can be fatal, so monitoring paraplegic sitting and lying pressure distribution is necessary [10]. The research on the human pressure distribution test system has been in development for more than 40 years and has led to a variety of products. The type of pressure distribution sensor can be divided into capacitor, piezoresistive and strain type. The capacitive pressure distribution test system is composed of cathode and anode. The distance between the capacitor plates will change under the pressure of the human body, and thus the dynamic change of the capacitance value, so the pressure distribution can be retrieved by measuring the change of the capacitance value. The mattress capacitive body pressure test system, developed by XSENSOR Company, is a more advanced human body pressure distribution test system [11,12,13]. This system is soft and durable, but the accuracy of the surface pressure distribution is reduced due to its thickness. Resistive human body pressure measurement systems are measured using reduced contact resistance between two layers of film when they are stressed. The main structure of the resistive pressure distribution sensor manufactured by INITERLINK is a two-layer adjacent polyester film [14]. It is made of a metal powder or a carbon black-impregnated composite. One layer of polyester film is a high-resistance conductive polymer sensitive film, known as the resistance ink. The other layer is printed on the polyester film with interdigitated expandable electrodes. This type of human body pressure distribution sensor is thin and flexible, has a low cost, and has simple circuitry, but its output is nonlinear, it has high power consumption, it is not durable, and it has a slow response. A piezoresistive human pressure sensor is a sensor that uses the piezoresistive effect of semiconductor material to convert measured pressure to an electrical signal output. The piezoresistive effect consists in the fact that, when stress occurs, resistivity will obviously change. Canada’s Vista Medical’s pressure distribution test system uses piezoelectric resistance pressure sensing technology [15]. The thickness of the system is only 0.36 mm. Due to the influence of temperature and humidity, the measured value drifts over time. The pressure distribution measurement system described above is based on the array type, requiring a large number of pressure measurement units. The resolution of the pressure distribution measurement system depends on the number of array units, which causes a complex circuit design, a high manufacturing cost, and a limited application range.

This paper introduces a human pressure distribution measurement system based on a conductive silica gel piezoresistive effect. In order to avoid allergic reactions, considering the biological characteristics of silica gel, the system uses conductive silica gel as a sensitive material. The pressure distribution of body pressure on the conductive silica membrane leads to a difference in conductivity distribution, and the distribution of conductivity can be obtained via electrical resistance tomography. In order to improve the quality of electrical impedance inversion and reduce the interference of electrode contact resistance on the electrode voltage, wavelet transform is proposed to remove this interference. High precision operational amplifier OP77 constitutes a high precision constant current source as the excitation constant current source of conductive silica. The measurement mode of the measuring electrode is controlled by the analog multiplexer CD 4067. Raspberry Pi is used as the main controller of the human body pressure distribution measurement system, and the wavelet transform and the resistance tomography algorithm are realized using Python. The experimental results show that the pressure distribution measurement method proposed in this paper can accurately measure the human pressure distribution. Compared with the traditional lattice pressure distribution measurement system, the method greatly reduces the number of electrodes. In addition, the scheme using Raspberry Pi as the main controller also lays the foundation for further function expansion of the pressure distribution measurement system.

## 2. System Hardware Architecture

**Figure 1:**
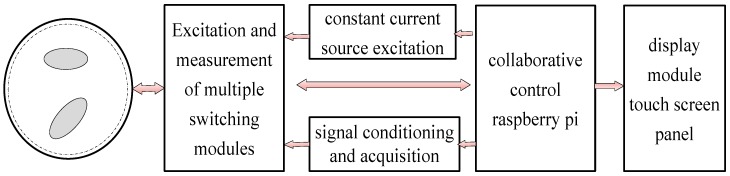
The diagram of the human pressure distribution acquisition system (HPDAS).

Figure 1 shows a schematic diagram of the human pressure distribution acquisition system (HPDAS) based on electrical resistance tomography.

The HPDAS includes five modules: a constant current source excitation module, a multiplex module, a signal conditioning and acquisition module, a collaborative control module, and a display module. In order to simplify the complexity of the inversion algorithm and improve the accuracy of inversion imaging, the excitation source of the conductive silica gel is a constant current source. Here, we used the positive feedback circuit composed of operational amplifier to establish a constant current source. The role of the multi-switch module is to complete the selection of excitation electrodes and acquisition channels. As shown in Figure 1, the system is a 16-electrode array, and the working mode is the current adjacent excitation, the electrode adjacent measurement mode. The automatic switching of the excitation and measurement electrodes is achieved by controlling the multi-resistance analog switch via the main control module. The 16-channel multiplexer is the node of the constant current excitation input and the voltage signal output of the electrode. The multiplexer performs the excitation input and the collector voltage in turn according to certain rules. The design of the circuit used in the type of 16-way multiplexer switch is CD4067. The main function of signal conditioning and data acquisition module is to amplify the weak voltage signal obtained by the measurement electrode. This part of the circuit mainly includes a pre-differential amplifier, a high-pass filter circuit, a digital–analog conversion circuit, and so on. The multiplexing module is connected to the conductive silicone sensor unit, and the signal conditioning and acquisition module is connected to the multiplexing module. The working process of the system is as follows: First, the main control module sends a signal to the excitation multiplexing module and turns on the excitation switch. The current generated by the constant current source module is energized by energizing the multiple selection control module into the corresponding electrode pair of the conductive silica gel force sensitive sensor. The main controller then sends a signal to the measuring multiplexer to measure the pair of electrodes to be connected to the differential amplifier input section. Digital-to-analog conversion using 24-bit chip and the type of chip is ADS1256. The role of the collaborative control module is to coordinate the modules that make up the system so that it can work together, specifically to coordinate the timing of the excitation, acquisition, amplification, storage, and display modules. Taking into account the ease of debugging in system development and system scalability, we chose Raspberry as the main controller. The role of the display module is to show the result of inversion imaging. Here, we used a Raspberry-dedicated 3.5-inch touch screen TFT display.

## 3. Measurement Mechanism

Silicone has a soft touch, which makes it very suitable for human body pressure sensing materials. The conductive silica gel is a composite conductive polymer by adding nano-carbon black in the silica gel. The material has a piezoresistive effect, and the resistivity of the area under pressure will decrease when the conductive silica film is under pressure. The technical principle of the human body pressure distribution system proposed in this paper is to calculate the body pressure distribution by measuring the conductivity of the conductive silica gel under different pressures. The pressure distribution information of the conductive silica gel can be obtained by the conductivity distribution of the sensitive field dielectric. A sensitive field can be established by injecting a constant current into the electrodes on the conductive silica boundary. When the distribution of the conductivity field in the sensitive field changes, the potential distribution in the sensitive field changes, resulting in the corresponding change of the measured voltage on the sensitive field boundary. The change of measurement voltage reflects the change information of electrical conductivity. Using the measurement voltage on the boundary, the electrical conductivity distribution in the sensitive field can be reconstructed by image reconstruction algorithm, so as to realize the visualization measurement of the human pressure distribution.

### 3.1. Sensing Mechanism of Conductive Silica

The material used in the human body sensor in this paper is conductive silica gel. Silica gel is a kind of biological material, which can simulate the long-term burial of human organs in the body to replace part of the function of a missing organ. For example, long-term indwelling products include hydrocephalus drainage devices, artificial lungs, retinal implants, larynxes, palm joints, artificial tympanic membranes, artificial heart valve accessories, and so on. Conductive silica gel only makes the nano-carbon powder into the silica gel. Nano-carbon powder is a non-toxic additive that is completely harmless to the human body.

The nanoparticles are infiltrated into the silica gel to form a current carrier in a conductive silica gel. The conductive nature of conductive silica is the directional movement of the current carrier in the electric field. The sufficient number of current carriers and the formation of the conductive channel between the two chains are two factors in conductive polymer conduction. The conductive silica gel will produce elastic deformation when it is under pressure, and the resistance decreases with the increase in pressure. This is mainly due to the increase in pressure. The particle spacing decreases, and the probability of particle contact increases. It is easier to form a conductive network and improve the conductive properties of the material. The piezoresistive calculation model of carbon-black-filled conductive silica gel polymer is as follows:

(1) $$\rho_{m}=\rho_{h}{\left(1-\phi_{c}\right)}^{\tau }{\left[\phi_{0}\exp\left(\frac{1-2\upsilon }{E}P\right)-\phi_{c}\right]}^{-\tau }$$

where *φ_c_* is the critical percolation threshold, and *τ* is the percolation coefficient of composites. *ρ*_h_ is the resistivity of carbon black particles, *φ*_0_ is the volume fraction of conductive silica without loading force, *E* is the elastic modulus of conductive silica gel, *v* is the Poisson's ratio, and *P* is the pressure.

### 3.2. Human Pressure Distribution Measurement System Based on Electrical Resistance Tomography

**Figure 2:**
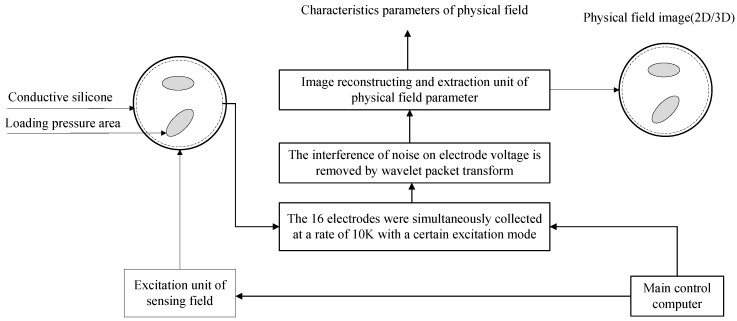
The process of visual inversion of pressure distribution.

The detailed measurement principle of the human body pressure distribution measurement system is shown in Figure 2. The material of the human pressure distribution measurement sensing film is conductive silica gel, which can be made into different shapes to meet specific requirements. In this paper, the sensing film is made circular to test its performance. Measurement and excitation electrodes are evenly distributed on the edge of the circular film.

The principle of electrical resistance tomography is to obtain information on medium distribution in the sensitive field by judging the resistivity distribution of the medium in the sensitive field according to the resistivity of different media. That is, when the current excitation is applied at the boundary of the sensitive field, the electric potential distribution in the sensitive field changes with the change in resistivity distribution in the sensitive field, resulting in a corresponding change in measurement voltage on the boundary of the sensitive field. The variation in the measured voltage reflects the change in resistivity. Using the measurement voltage on the boundary, the resistivity distribution in the sensitive field can be reconstructed by a certain image reconstruction algorithm, so as to realize the visualization measurement.

The electrodes arranged on the edge of the conductive silicone are numbered 1 to 16. These electrodes serve both as a measuring electrode and as an excitation electrode, and their functions can be controlled by multiple switches. The excitation electrode mode is selected for the adjacent mode. The adjacent excitation mode applies an excitation current to two adjacent electrodes, measures the voltage on two adjacent electrodes of the non-excitation electrode, and then repeats the above process until all of the adjacent electrode pairs are excited.

**Figure 3:**
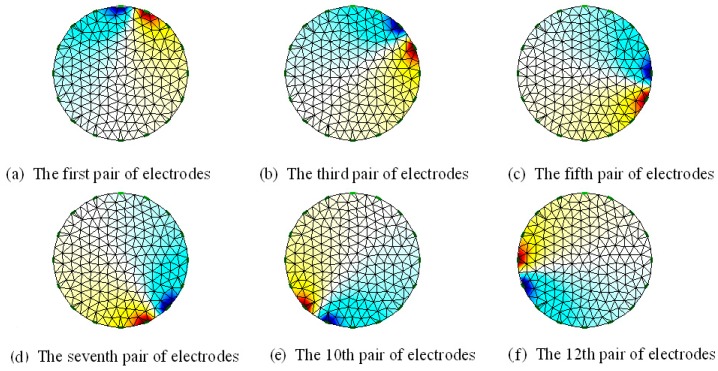
The voltage pattern from adjacent mode.

Figure 3 shows the potential distribution at which a different electrode pair is injected with a current of 10 mA when the conductive silica gel is not pressed in the adjacent excitation mode. The solution to the results of Figure 3 is the forward process of resistance tomography (ERT). Its solution principle is based on the approximate assumption of the following two facts: (1) The sensitive field of the ERT is an approximate steady-state field. When energization is applied at the boundary of the field, the electric field changes at the same time, ignoring the time that the current travels from one point to another in the electric field. (2) The ERT-sensitive field is a constant electric field, and there is no current source or current sink in the excitation field, so the divergence of the current in the sensitive field is zero. According to the above two assumptions and the Maxwell equation of constant electric field, we can obtain a mathematical description of ERT forward problem:

(2) $$\left\{ \begin{array}{c} \nabla \cdot \left(\sigma \cdot \nabla \phi \right)=0\;\;\;\;\;\;\;\;\;\;\;\;\;\;\;\;\;\;\;\;\;\;\;\;\;in\;\Omega \;\\ {\int^{\text{​}}}_{S1}\sigma \frac{\partial \phi }{\partial n}ds=+I\;\;\;\;\;on\;source\;electrode\\ {\int^{\text{​}}}_{S2}\sigma \frac{\partial \phi }{\partial n}ds=-I\;\;\;\;\;on\;sink\;electrode\\ {\frac{\partial \phi }{\partial n}|}_{S3}=0\;\;\;\;\;\;\;\;\;\;\;\;\;\;\;\;\;\;\;\;\;\;\;\;\;\;\;\;\;\;others\; \end{array}\right.$$

where ϕ is the potential distribution, σ is the conductivity distribution, S1 and S2 are the surface of the current injected electrode and the current out of the electrode, S2 is the insulating surface of the conductive silicone. At present, the method of solving Equation (1) is mostly solved by the finite element method, and the software solution technology of the finite element is very mature. OpenFOAM (open field operation and manipulation) is a free tool that can simulate the calculation of electromagnetic fields. OpenFOAM can be used to model ERT forward problems. A conductive silicone sensor with N electrodes M=N(N−3)/2 independent voltage values can be measured using the adjacent excitation mode. We can obtain M equations similar to Equation (1), as well as its discretization and linearization. The ERT positive problem of the observation model is

(3) U=S·G

In Equation (3), U is the normalized measurement electrode voltage vector, S is the normalized sensitivity matrix, and G is the normalized conductivity. The key step in ERT human body pressure distribution image reconstruction is to solve the inverse problem, which is to obtain the conductivity distribution by the measured M voltage. In short, how do we get the value of G in Equation (3)?

The pressure distribution image inversion process has two main difficulties. One is the problem of obtaining the sensitivity matrix. In the sensitive field, the sensitivity distribution is not uniform, which is easily affected by the distribution of the media, that is, the so-called “soft field” effect; how to obtain the sensitivity matrix effectively and accurately has an important influence on the imaging quality. The other is to reduce the interference of noise to the electrode measurement voltage. Equation (3) is an ill-conditioned systems of equations. Its solution is unstable, and the small perturbation of voltage value U will have a great influence on the image gray value G.

**Figure 4:**
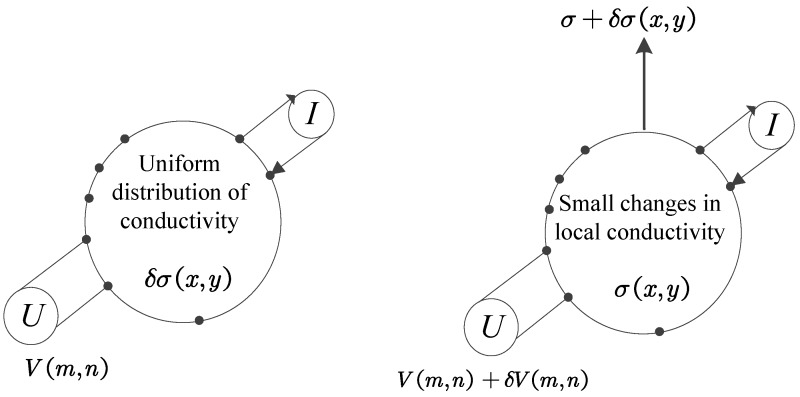
Four-electrode current excitation voltage measurement method.

The sensitivity coefficient is related to the shape and conductivity of the conductive silica gel, which can be calculated via finite element analysis. The physical significance of sensitivity is illustrated by typical four-electrode current excitation voltage measurement, as shown in Figure 4.

Assuming that the conductivity in the field, σ, is uniformly distributed, the excitation current *I* is injected into the *m*th pair of electrodes, and the voltage measured V(m,n) on the *n*th pair of electrodes. When the conductivity of a certain region changes slightly δσ(x,y), the measured voltage on the corresponding *n*th electrode changes to V(m,n)+δV(m,n). If δ(x,y) is small enough, it can be assumed that the distribution of the equipotential line in the sensitive field is unchanged before and after the change of the conductivity, and the variation of the measurement voltage is proportional to the change in conductivity. The proportionality constant is defined as the sensitivity coefficient $S_{m,n,x,y}$:

(4) $$S_{m,n,x,y}=\frac{\delta V\left(m,n\right)}{\delta \sigma \left(x,y\right)}$$

According to Gese–Lowitz compensation [16] and the finite element principle, Equation (4) can be converted to

(5) $$S_{i,j}=\frac{1}{2A_{ej}I_{n}I_{m}}{\left[\varphi_{mj}^{k}\right]}^{T}\left[Y_{j}\right]\left[\varphi_{nj}^{k}\right]$$

where m is the serial number of the excitation electrode pair, and *n* is the serial number of the measurement electrode pair. The subscript *i* represents the *i*th independent measurement, $e_{j}$ denotes the jth unit, $\varphi_{n},\varphi_{m}$ are the potential distributions of the nodes in the field, respectively, when the excitation current $I_{m},I_{n}$ is injected into the *m*th and nth pairs of electrodes. $A_{ej}$ is the area of the *j*th unit. $\varphi_{mj}^{k},\varphi_{nj}^{k}$ is the potential of k nodes on the corresponding unit $e_{j}$. $Y_{j}$ is the finite element coefficient matrix of element $e_{j}$.

**Figure 5:**
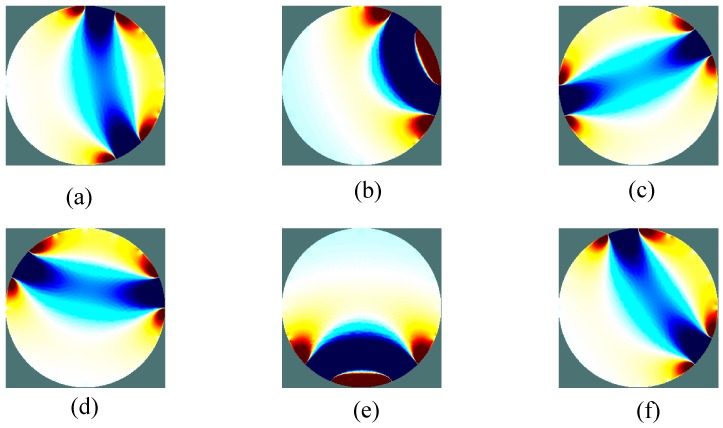
The Sensitivity field of Electrically Conductive Silicone. (**a**) is the sensitivity field distribution of silicone rubber between electrode 1 and electrode 7; (**b**) is the sensitivity field distribution of silicone rubber between electrode 2 and electrode 4; (**c**) is the sensitivity field distribution of silicone rubber between electrode 3 and electrode 11; (**d**) is the sensitivity field distribution of silicone rubber between electrode 4 and electrode 14; (**e**) is the sensitivity field distribution of silicone rubber between electrode 6 and electrode 9; (**f**) is the sensitivity field distribution of silicone rubber between electrode 5 and electrode 15.

Figure 5 is a sensitive field distribution of conductive silicone as a sensor for human pressure distribution. For the convenience of image inversion, the shape of the sensor of the conductive silicone pressure distribution test system is circular. Sixteen electrodes are evenly arranged around the outer periphery of the circular conductive silica gel. There are a total of 208 measurement models. The size of the sensitivity matrix is  208 × 5093.

In principle, after obtaining the sensitivity matrix, the distribution of electrical conductivity G can be distributed by measuring the electrode voltage in various excitation modes. However, Equation (3) is an ill-conditioned systems and very sensitive to voltage measurements, which must be accurate so as to remove measuring voltage signals containing noise, so that a good conductivity distribution image can be obtained.

### 3.3. Wavelet Transform and Noise Removal

Wavelet transform is the expansion and approximation of a function on wavelet basis in a specific space. It is the perfect crystallization of functional analysis, Fourier analysis, spline analysis, harmonic analysis, and numerical analysis. From an engineering point of view, wavelet analysis is a signal and information processing tool, is another effective time–frequency method in addition to Fourier analysis, can be used in time domain and frequency domain analysis, and has time–frequency localization and multi-resolution characteristics. Therefore, it is known as the “digital microscope” of the analysis signal, especially suitable for dealing with non-stationary signals.

Definition: If $\psi (t)\in L^{2}(R)$ and $\overset{\wedge }{\psi }(0)=0$, then

(6) $$\psi_{a,b}(t)={\left|a\right|}^{\frac{1}{2}}\psi (\frac{t-b}{a}),b\in R,\;\;a\in R-\{0\}$$

This is a continuous wavelet, where ψ(t) is the basic wavelet or generating wavelet in function space $L^{2}(R)$, t is the variable in real number field R, *a* is the scale or location coefficient, and *b* is the translation factor, and satisfies the admissible condition

(7) $$C_{\psi }=\int_{-\infty }^{+\infty }\frac{{\left|\overset{\wedge }{\psi (\omega )}\right|}^{2}}{\omega }d\omega <\infty$$

where $\overset{\wedge }{\psi }$ is the Fourier transform of ψ(t) at frequency ω.

If a signal f(t) is deconstructed into the function set, the continuous wavelet transform $\left(W_{\psi }f\right)$ is defined as

(8) $$(W_{\psi }f)(a,b)=<f,\psi_{a,b}>={\left|a\right|}^{\frac{1}{2}}\int_{-\infty }^{+\infty }f(t)\bar{\psi }(\frac{t-b}{a})dt$$

where “< >” indicates the inner product, $\left(W_{\psi }f)(a,b\right)$ are the coefficients of wavelet transform corresponding to scale and location (a and b), and $\bar{\omega }$ represents the conjugations of ω. This transform is known as a continuous wavelet transform (CWT) when *a* and *b* are continuously changing, and as a discrete wavelet transform (DWT) when *a* and *b* are discrete points.

Typically, *a* and *b* are taken as a power series:

(9) $$a=a_{0}^{j};\;\;\;b=ka_{0}^{j}b_{0};\;\;j,k\in Z$$

when $a_{0}=2\;\mathrm{and}\;b_{0}=1$, the scale and translation are dyadic discrete and the dyadic wavelet is obtained:

(10) $$\psi_{j,k}(t)=2^{\frac{j}{2}}\psi (2^{-j}t-k)$$

Hereafter, the DWT is represented as this dyadic wavelet transform, and the DWT coefficients are

(11) $$\psi_{j,k}(t)=2^{\frac{j}{2}}\psi (2^{-j}t-k)$$

$c_{j,k}$ is the kth largest value of the coefficients in the *j*th deconstruction level.

The reconstruction formula is

(12) $$f(t)=\sum_{j\in Z}\sum_{k\in Z}c_{j,k}\psi_{j,k}(t)$$

The basic idea of wavelet transform de-noising can be summarized as follows: the noise signal of the electrode voltage signal is decomposed into multi-scale by wavelet transform. The wavelet transform is used to divide the wavelet coefficients, and the wavelet coefficients are then removed at each scale. The wavelet coefficients belonging to the signal are enhanced, and the signal after wavelet de-noising is finally reconstructed. The question to answer is, by what criterion can the wavelet coefficients of the noise be removed, and what part of the measurement electrode voltage signal be enhanced? In this method, the median filter based on the wavelet is used to de-noise. Median filtering is a commonly used non-linear method to suppress noise and can overcome the problem of signal mutation caused by linear filtering, such as least mean square filtering and mean filtering, so as to obtain a more satisfactory de-noising effect. The principle is very simple: A window with a length of N is moved in the time axis of the voltage signal. In each position, the voltage value in the window is arranged from so small to large, and the intermediate value is used as the output value of the window. One of the biggest advantages of wavelet transform is that the wavelet function family is very rich and can have many choices. Wavelets generated by different wavelet coefficients have different effects in removing noise. Noise is often manifested as a sudden change in electrode voltage amplitude, with high frequency characteristics and time irrelevance. The electrode voltage signal is decomposed by the wavelet to obtain the low frequency part and the high frequency part. The low frequency part reflects the outline of the signal. The high frequency part is reflected in the details of the signal and the noise is mixed. Therefore, the de-noising of the electrode voltage signal only needs to deal with its high frequency coefficients. The specific noise removal steps are as follows:

- Step 1: A set of wavelet coefficients $\omega_{j,k}$ is obtained by making wavelet transform of electrode signal $V_{i}\left(t\right)$ with noise
- Step 2: Given a default wavelet decomposition level *j*, and coefficients of j+1 and higher, all coefficients are retained.
- Step 3: For the wavelet decomposition coefficient of the layer i(1<i<j), the $n_{i}$ coefficient of the maximum absolute value is kept, and the value of $n_{i}$ is determined by the following formula:
  (13) $$n_{i}=M{\left(j+2-i\right)}^{\eta }$$

In this formula, M and η are empirical coefficient, where the default is M=L(1), which is the length of the coefficient. M satisfies the inequality L(1)≤M2≤L(1), and the value of general condition η is 3.

- Step 4: According to the threshold of the wavelet decomposition coefficients determined in Step 3, the wavelet coefficients greater than the threshold value are set to zero.
- Step5: Using $\omega_{j,k}$ to reconstruct the wavelet, we obtain the estimated signal ${\hat{V}}_{i}\left(t\right)$, that is, the electrode voltage signal to remove the noise.

After obtaining accurate measurement of the sensitive field coefficient and the electrode voltage, the resistance tomography technique can be used to solve the distribution of the sensitive field conductivity. The implementation steps of the algorithm are as follows:

- Step 1: The ERT sensitive field is divided and the solution domain is discretized.
- Step 2: The coverage matrix of the equipotential of the projection area is generated, the sensitive field is divided into several equipotential regions, and the equipotential regions corresponding to each cell are determined.
- Step 3: The boundary measurement voltage is solved by the ERT system or the finite element method.
- Step 4: The back projection algorithm is used to perform back projection imaging and display imaging results.

## 4. Results

**Figure 6:**
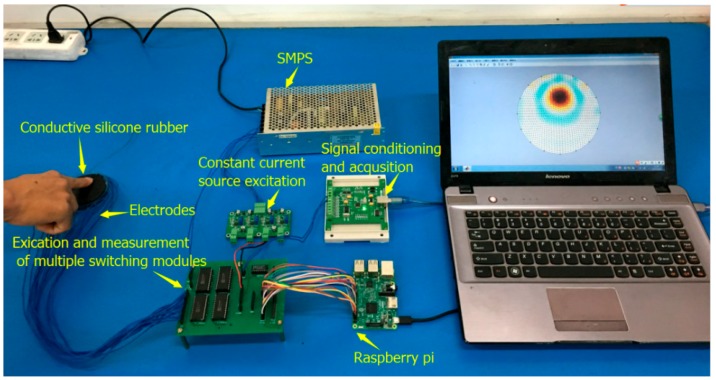
Experimental device of pressure distribution imaging system.

Figure 6 shows the experimental device for testing human pressure distribution based on the resistivity tomography of conductive silica gel. The power supply of the entire device uses a multi-voltage output switching power supply for the experimental device of different chips to provide voltages of ±5 V and ±12 V. The constant current source excitation module provides the constant current excitation signal for the conductive silica gel. The ideal current source of the constant current source should have a characteristic that does not change due to changes in load and ambient temperature. The change of the resistance caused by the pressure on the conductive silica will be mapped to the variation in the voltage between the electrodes under the excitation of the constant current source.

The sensor of the human pressure distribution test device is a circular conductive silica gel with a diameter of 65 mm and a thickness of 12 mm. The electrode is a copper sheet with a diameter of 3 mm. Sixteen copper electrode pieces of the same size, equally spaced on the outside of the cylindrical conductive silicone, form a sensitive electrode array. The sensitive electrode array is connected to the multiplexer module via 16 cables. We used Raspberry Pi to control the multi-channel electronic switch so as to achieve the excitation and measurement of the electrode automatically switch. The sequential input of the excitation signal and the voltage signal are connected by the 16-channel switch and the electrode pair. The signal conditioning and acquisition module is used to collect the voltage signal between the electrodes. Because the voltage difference between the conductive silica electrodes induced by constant current excitation is the mv level, it is firstly filtered and amplified by the conditioning circuit. In order to acquire the micro change of conductivity of the conductive silica gel, a 24-bit high-precision digital–analog conversion chip (ADS1256) is used.

**Figure 7:**
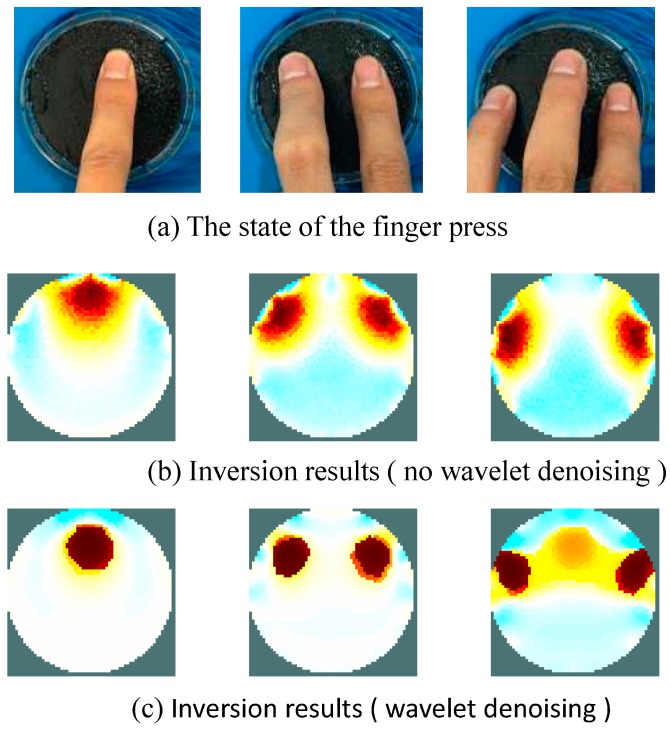
Experimental results of pressure distribution imaging.

As shown in Figure 7, the conductive silica gel of the human body pressure test device was finger-pressed. The experimenter pressed the conductive silica gel using the middle finger, the index finger and the middle finger, and finally the ring finger, index finger, and the middle finger. Figure 7b,c show the analysis and inversion using the human pressure distribution collection and analysis device presented in this paper. Figure 7b shows the inversion results without wavelet de-noising. Figure 7c shows the effect of inversion imaging after de-noising with wavelet. It can be seen from Figure 7 that the pressure distribution inversion imaging effect is significantly improved after wavelet de-noising. The removal of noise from the voltage signal of the electrode was realized by software, solving the problem of high hardware costs in the process of noise removal. In addition, the use of the wavelet de-noising scheme can be optimized according to the application environment and occasion.

## 5. Conclusions

In this study, we propose a pressure distribution sensing system based on wavelet analysis and resistance tomography to overcome the shortcomings of traditional dot matrix electrode pressure distribution acquisition system, which require multiple electrodes and have a high production cost. The system uses ADS1256, a conductive silicone sensor layer, a constant current source module, a serial communication module, a Raspberry Pi host, a touch screen, and other components. We achieved the following: (1) The wavelet transform was used to pretreat the collected electrode voltage signal and improve the anti-jamming performance of the system. (2) The resistance tomography method was used to realize the real-time imaging of the pressure distribution, which reduces the disadvantages of the traditional dot matrix pressure distribution sensor, which requires a large number of electrodes and inconvenient maintenance. (3) Raspberry Pi was used as the main controller of the program, which solves the networking function, can be used for future upgrades, and can facilitate functional expansion.
